# Supplementary material for: Transcriptome Analysis Reveals the Mechanism by Which Exogenous Melatonin Treatment Delays Leaf Senescence of Postharvest Chinese Kale (Brassica oleracea var. alboglabra)
Source: Int J Mol Sci. 2024 Feb 13;25(4):2250. doi: 10.3390/ijms25042250 (PMC10889248; doi:10.3390/ijms25042250)
Supplement: Supplementary file 1 [file ijms-25-02250-s001.zip › Di et al. Supplementary Figure S1.pdf]

Supplementary Figure S1

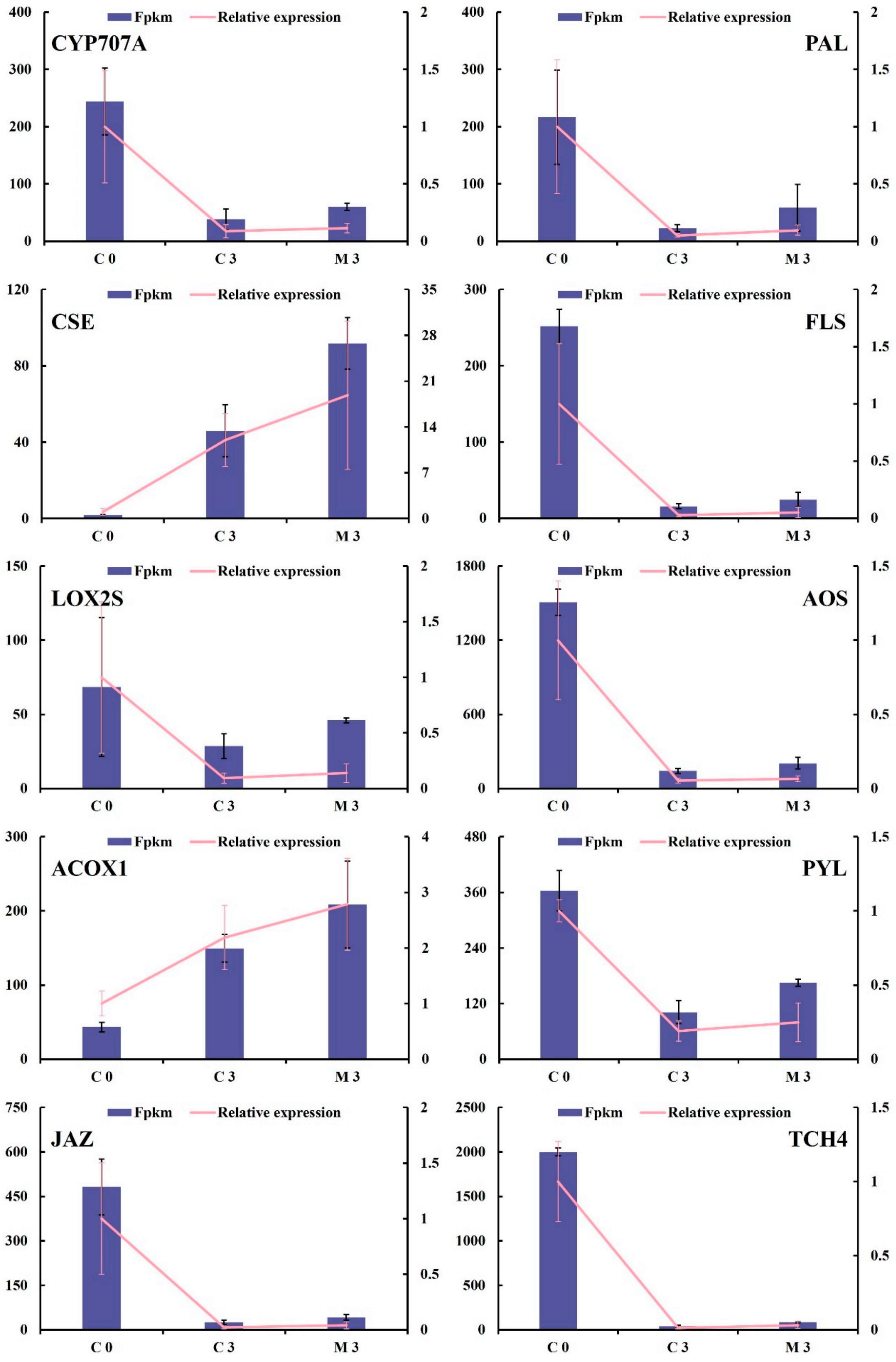

Supplementary Figure S1 Expression pattern of ten selected DEGs obtained by RNA-Seq and qRT-PCR.
